# Supplementary material for: Thrombin Preconditioning Improves the Therapeutic Efficacy of Mesenchymal Stem Cells in Severe Intraventricular Hemorrhage Induced Neonatal Rats
Source: Int J Mol Sci. 2022 Apr 18;23(8):4447. doi: 10.3390/ijms23084447 (PMC9030410; doi:10.3390/ijms23084447)
Supplement: Supplementary file 1 [file ijms-23-04447-s001.zip › ijms-1657970-supplementary.pdf]

International Journal of
Molecular Sciences

MDPI

Supplementary Materials

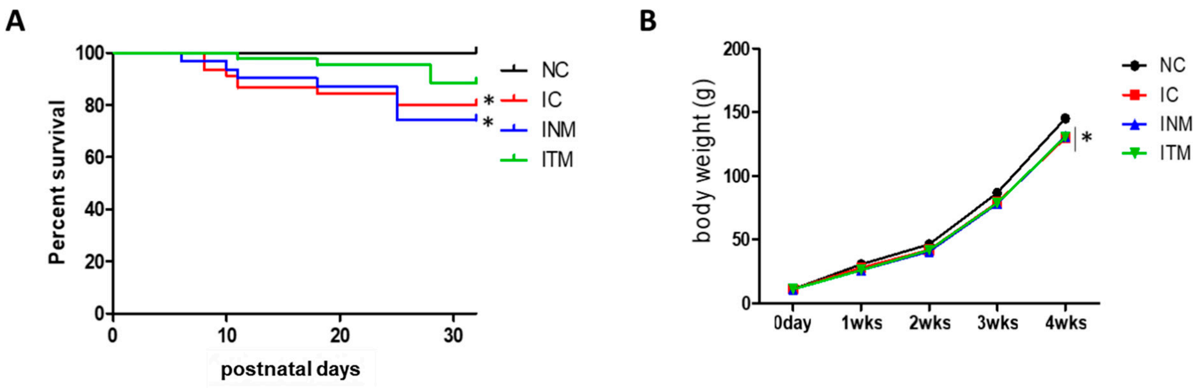

Figure S1. (**A**) Survival rate and (**B**) body weight growth curve {initial number of animals (-the number of dead animals) = 18 (-0), 36 (-7), 31 (-8), 32 (-4) in the NC group, IC group, INM group and ITM group, respectively}. The measured values are expressed as mean ± SEM. *$p < 0.05$ versus NC. NC, normal control; IC, IVH control; INM, IVH with transplantation of naïve MSCs; ITM, IVH with transplantation of thrombin preconditioned MSCs.

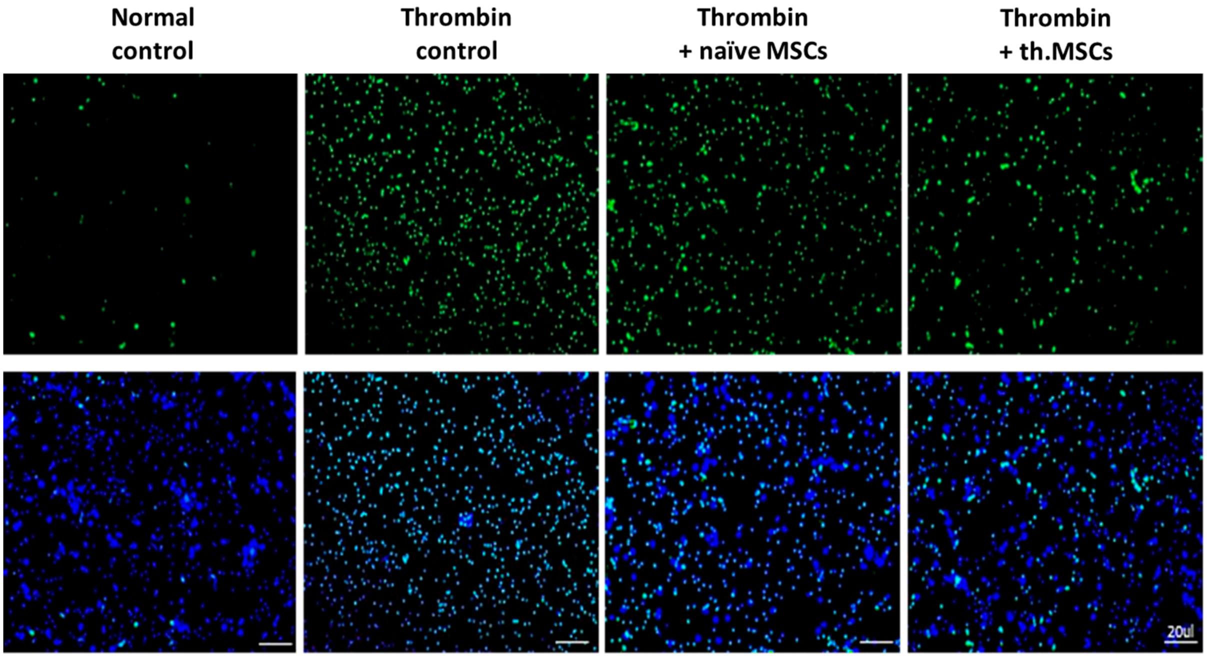

Figure S2. Enlarged images shown in Figure 1D.
